# Primary rectal melanoma in an African female: a case report

**DOI:** 10.11604/pamj.2022.41.286.33128

**Published:** 2022-04-07

**Authors:** Werimo Pascal Kuka, Joseph Gatheru, Sitna Mwanzi, Nelson Onyango, Allan Rajula

**Affiliations:** 1 Department of Medicine, Aga Khan University Hospital, Third Parklands Rd, Nairobi, Kenya,; 2 Department of Pathology, Aga Khan University Hospital, Third Parklands Rd, Nairobi, Kenya,; 3 Department of Hematology and Oncology, Aga Khan University Hospital, Third Parklands Rd, Nairobi, Kenya,; 4 The Ohio State University College of Medicine, Columbus, Ohio, USA

**Keywords:** Rectal, malignant melanoma, rectal cancer, Kenya, case report

## Abstract

Primary malignant melanoma occurs frequently on the skin and is rare in people of African ancestry. The rectal region is an unusual site for non-cutaneous melanoma. We report a case of a 58-year- old African woman presenting to a Kenyan hospital with lower abdominal pain and per rectal bleeding for three months, who underwent a colonoscopy that showed a rectal polypoid mass at the anorectal region. Histology of the mass showed pigmented pleomorphic cells which had positive stains for melanoma markers. Staging workup performed, including magnetic resonance imaging (MRI) of the pelvis and positron emission tomography (PET) - computed tomography (CT), showed regional lymph node involvement but no evidence of distant metastases. Surgery was recommended to the patient but she died eight months after the diagnosis. The case illustrates that primary rectal melanoma, though rare in Africans, is an aggressive disease which can be easily misdiagnosed as hemorrhoids or rectal adenocarcinoma.

## Introduction

Rectal melanoma is a rare and aggressive malignancy accounting for less than 0.05% of colorectal tumors and only 2% of melanomas [1]. The rectum is the third most common site for non-cutaneous melanomas after the head and neck region and the female genitourinary tract [2]. The median age at diagnosis is the seventh decade and the incidence is higher in females [1,3,4]. Primary rectal melanoma portends a poor prognosis, with a current 5-year survival rate of between 12-20% [1,2,5,6].

Cutaneous melanoma mostly occurs in sun exposed areas and is more common in Caucasians than in people of African or Asian ancestry. The occurrence of the malignancy in the non-sun exposed area of the rectum is thought to originate from the melanocytes in the rectal epithelium [7]. The optimal treatment modality for rectal melanoma is surgical resection. Chemotherapy and radiation therapy have not shown any benefit in the management of rectal melanoma and few studies have assessed the growing role of targeted therapy and immune checkpoint inhibition in the treatment of mucosal melanoma [5,8]. Due to the rarity of rectal melanoma, there are few published cases from the Sub-Saharan Africa region. This case illustrates a diagnosis and outcome of rectal melanoma in an African woman presenting to a tertiary Kenyan hospital.

## Patient and observation

**Patient information:** a 58-year-old female presented with complains of episodic lower abdominal pain and per rectal bleeding for three months. She denied any weight loss and had no family history of cancer. Her medical history was not significant.

**Clinical findings:** a digital rectal examination revealed a mass at the 3 o´clock position. The examining finger was blood stained.

**Figure 1:**
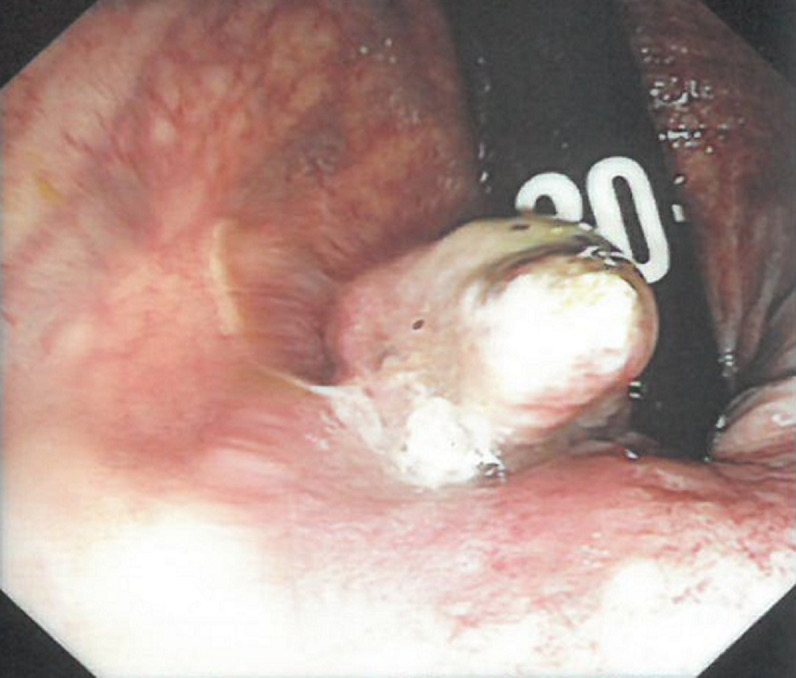
colonoscopy revealed a mass at the anorectal junction

**Figure 2:**
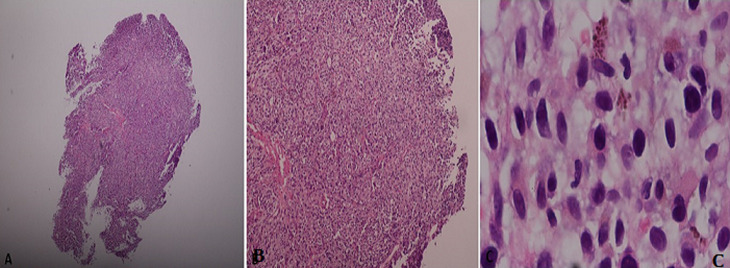
hematoxylin and eosin x 1000 a) lesion comprising moderately pleomorphic cells containing Brown granular cytoplasmic inclusions original magnification x 1000)

**Figure 3:**
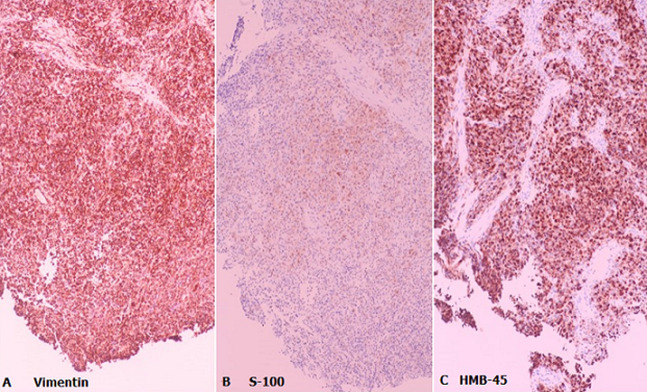
immunohistochemistry stains x 200, cytoplasm of the malignant cells stained positive for vimentin (A), focally positive with antibodies against S-100 (B) and strongly positive with antibodies against HMB-45 (C)

**Diagnostic assessment:** a colonoscopy was performed with findings of an eccentric and friable mass at the anorectal region (Figure 1) which was biopsied. There was no obstruction of the lumen. On microscopy, fragments appeared to be composed of sheets of large pleomorphic cells and only a small focus of residual normal colonic mucosa was seen. The pleomorphic cells were characterized by large nuclei, prominent nucleoli with some having brown granular pigment in the cytoplasm (Figure 2). Immunohistochemistry was positive for melanoma markers human melanoma black 45 (HMB-45), S - 100 and Vimentin (Figure 3).

**Figure 4:**
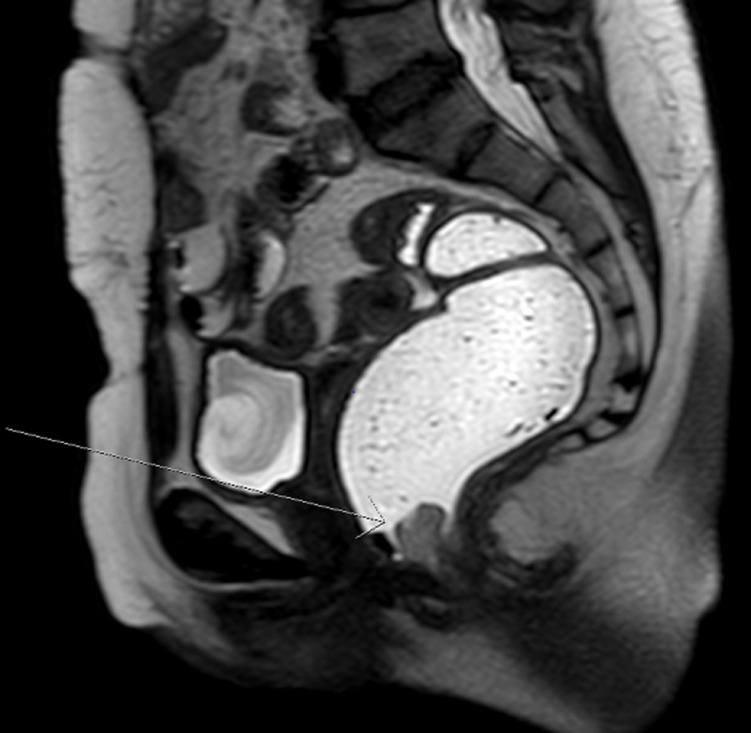
MRI pelvis, sagittal T2 weighted image showing lobulated enhancing mass arising from the lower rectum close to the anal sphincter

**Diagnosis:** a diagnosis of malignant melanoma was established. BRAF V600 mutation analysis was negative. Staging workup done included MRI pelvis and whole body PET-CT scan. The MRI pelvis revealed a 1.7 cm x 1.2 cm mildly lobulated intensely enhancing mass arising from the lower rectum close to the anal sphincter at the left lateral position (Figure 4). The polypoid endoluminal mass was involving the internal sphincter but not extending to the external sphincter. Several left mesorectal lymph nodes were seen, the largest measuring 8 mm. The PET-CT scan showed a metabolically active rectal tumor with regional spread but no evidence of distant metastasis.

**Therapeutic interventions:** a multidisciplinary tumor board recommended abdominoperineal resection as the modality of treatment. However, the patient declined any intervention.

**Follow-up and outcome on interventions:** the patient died eight months later at a hospice facility.

**Patient perspective:** before being referred to the gastroenterology clinic, I had been told that the cause of bleeding could be hemorrhoids. However, after the colonoscopy showed a growth, the doctors informed me that it was cancer. They recommended surgery but I was not for it.”

**Informed consent:** written informed consent was obtained from the patient for publication of this case report and any accompanying images after the initial diagnosis. The family members (husband and daughter) were contacted after the death of the patient and also gave consent for the publication.

## Discussion

Rectal melanomas are rare and aggressive malignancies with poor outcomes. Most information regarding the condition is from case reports but cohort analysis have also established the epidemiology and prognosis. The reported case series have largely been from databases and hospital records of European and North American national cancer institutes. To our knowledge, no case of anorectal melanoma has been documented in Sub-Saharan Africa.

Melanoma within the anal region can occur proximal, at, or distal to the anal verge. Malignancies occurring proximal to the anal verge are termed as rectal while those distal to it are referred to as anal. Anorectal melanoma occur at the anal verge. The presentation is similar regardless to the site of the disease. The most common symptom is per rectal bleeding, reported by up to 55% of patients [3]. Other presentations include rectal pain, altered bowel habits and pruritus [5]. Due to the similarities in presentation, cases of anorectal melanoma are initially misdiagnosed as hemorrhoids [3]. The average duration of symptoms prior to diagnosis is 4-6 months [5]. Our patient had experienced per rectal bleeding and lower abdominal pain for three months prior to presentation for care and subsequent diagnosis.

The diagnosis of reported cases relied on endoscopy of the rectum, biopsy and histology. A diagnosis is established on histology by staining for melanin and immunohistochemistry, with positive staining for melanocyte markers including S100, HMB-45 and Vimentin [5]. All the three markers were positive in our patient. She had a negative analysis for BRAF V600 mutation. The mutation has been reported in 56% of cutaneous melanomas and 31% of all melanoma [9]. The frequency of BRAF mutation in melanoma in the rectal region has not been fully established. Edwards *et al*. reported zero cases of mucosal melanomas with BRAF mutations after analysis of thirteen specimens [10].

Staging of the extent of disease involvement can involve multiple radiologic methods. Endoscopic ultrasound has been advocated as a tool in assessment of tumor depth [5]. CT scan of head, neck, abdomen and pelvis can reveal the likely sites of metastases. Using these modalities plus abdominal ultrasonography, Belli *et al*. reported 42.5 % of patients having local disease while 27% had distant metastases at diagnosis [3]. In addition, MRI can be utilized to evaluate the depth, nodal status and local involvement whereas PET scan is useful for evaluation of indeterminate lesions [5,6].

The treatment of rectal melanoma is mainly surgical. Previous attempts at either chemotherapy or radiotherapy have been largely unsuccessful [5]. The optimal mode of surgery is still under debate. The approaches can be extensive resection by abdominoperineal approach or local resection with colonic end to anal anastomoses [4]. Belli *et al*. showed that there was no significant difference in overall survival in either radical or limited surgery [3]. Although Bullard *et al*. demonstrated that either surgical option is associated with similar rates of recurrences, they advocated for local wide excision due to reduced postoperative complications [4]. Regardless of the choice of surgery, the overall prognosis of rectal melanoma remains poor, with reported survival time of 15 months [1].

Targeted therapy and immune check point inhibition may play a role in advanced mucosal melanoma. Overall, the frequency of mutations in mucosal melanomas is lower than in cutaneous disease therefore lessening the efficacy of these modes of treatment [8]. Therapy targeting BRAF/MEK and KIT in advanced mucosal melanoma has been utilized in small cohorts of patients with varying results [8]. Immune checkpoint inhibitors, anti PD-1/PDL-1 and anti-CTLA-4 agents, have also shown promise in mucosal melanoma but larger studies need to be undertaken for conclusive results [8]. It remains to be seen if a combination of targeted therapy and immune checkpoint inhibition will have a role in treatment of advanced mucosal melanoma.

## Conclusion

While the incidence of rectal melanoma is low, it has a poor outcome and should be considered as a differential diagnosis in patients presenting with rectal bleeding and appropriate investigations including colonoscopy and biopsy with special stains should be done to rule it out.
